# Prediction of postoperative delirium after cardiac surgery by the interplay between preoperative plasma p-tau181 and IL-6 and heart-brain axis related factors: results from the prospective observational study FINDERI

**DOI:** 10.1038/s41380-025-03412-3

**Published:** 2025-12-22

**Authors:** Niels Hansen, Clara Maria Knopp, Hermann Esselmann, Christopher M. Celano, Carlotta Derad, Thomas Asendorf, Mohammed Chebbok, Stephanie Heinemann, Ihtzaz Malik, Barbara Morgado, Matilda-Marie Becker, Irina Günther, Iryna Krasiuk, Katharina Packroß, Alina Isabel Rediske, Nicholas Paul Süttmann, Tobias Titsch, Ingo Kutschka, Hassina Baraki, Jens Wiltfang, Christine A. F. von Arnim, Monika Sadlonova, FINDERI investigators, Niels Hansen, Hermann Esselmann, Christopher M. Celano, Carlotta Derad, Thomas Asendorf, Mohammed Chebbok, Stephanie Heinemann, Ihtzaz Malik, Matilda-Marie Becker, Irina Günther, Iryna Krasiuk, Katharina Packroß, Alina Isabel Rediske, Nicholas Paul Süttmann, Tobias Titsch, Ingo Kutschka, Hassina Baraki, Jens Wiltfang, Monika Sadlonova, Adriana Wiesent, Anke Jahn-Brodmann, Anne-Catherine Thiel, Annika Gaß, Barbara Morgado, Björn Hendrik Schott, Britta Albert, Charlotte Eberhard, Christine A. F. von Arnim, Clara Maria Knopp, Frederike E. Bauer, Jessica Schmitz, Julia Ehrentraut, Maike Hohberg, Manuel J. Santander, Maria Alexy, Maria Dornieden, Maria Knierim, Marianne Richter, Melania Hotheit, Michael Moser, Miriam F. Schröder, Paul T. Itting, Rebecca Arentz, Sandra Kastenbauer, Santander Martinez, Manuel Johannes, Sophie Doerfler, Tendai Chiwakata, Theresa Maria Kruck

**Affiliations:** 1 Department of Psychiatry and Psychotherapy, University Medical Center Göttingen, Georg-August-University, Göttingen, Germany; 2 Department of Geriatrics, University Medical Center Göttingen, Georg-August-University, Göttingen, Germany; 3 Department of Psychiatry, Massachusetts General Hospital, Boston, MA USA; 4 Department of Psychiatry, Harvard Medical School, Boston, MA USA; 5 Department of Medical Statistics, University Medical Center Göttingen, Georg-August-University, Göttingen, Germany; 6 Department of Cardiology and Pneumology, University Medical Center Göttingen, Georg-August-University, Göttingen, Germany; 7 Department of Cardiovascular and Thoracic Surgery, University Medical Center Göttingen, Georg-August-University, Göttingen, Germany; 8 DZHK (German Center for Cardiovascular Research), partner site Göttingen, Göttingen, Germany; 9 German Center for Neurodegenerative Diseases (DZNE), Göttingen, Germany; 10 Neurosciences and Signaling Group, Institute of Biomedicine (iBiMED), Department of Medical Sciences, University of Aveiro, Aveiro, Portugal; 11 Department of Psychosomatic Medicine and Psychotherapy, University Medical Center Göttingen, Georg-August-University, Göttingen, Germany

**Keywords:** Biological techniques, Predictive markers

## Abstract

Postoperative delirium (POD) following cardiac surgery is a severe complication. There is evidence of a link between neuroinflammation and neurodegeneration in POD. We investigated the preoperative proinflammatory interleukin-6 (IL-6) and neuronal damage marker phosphorylated tau protein 181 (p-tau181) to POD while considering preoperative heart-brain axis related factors. The prospective FINd DElirium RIsk factors (FINDERI) is an observational study in patients undergoing cardiac surgery. Biomarkers IL-6 and p-tau181 were measured in blood samples. For statistics, we utilized multiple logistic regression analyses and advanced machine learning techniques. In 491 patients, 106 (21.6%) developed POD. The age of patients with POD was significantly higher than that of patients without POD (*p* < 0.001). Preoperative IL-6 and p-tau181 levels independently predicted POD [IL-6: area under the curve (AUC) = 0.605, *p* < 0.005; p-tau181: AUC = 0.641, *p* < 0.0001)]. A multiple logistic regression analysis of preoperative log-transformed biomarkers levels (p-tau181, IL-6), female sex and cognitive performance increased the AUC (0.710, *p* < 0.0001) in predicting POD. We created a decision tree prediction model including preoperative p-tau181, IL-6, and the severity of mitral valve disease (training data: AUC = 0.672, *p* < 0.0001; validation data: AUC = 0.642, *p* < 0.05). The LASSO regression showed an increased AUC in the training (0.751, *p* < 0.0001) and validation dataset (0.652, *p* < 0.05). Our results demonstrate that the combined assessment of preoperatively measured p-tau181 and IL-6, preoperative mitral valve disease, cognitive performance and female sex, significantly predicts POD. These findings provide evidence that neuroinflammation and neuronal cell damage are associated with POD.

## Introduction

Postoperative delirium (POD) is a frequent complication in cardiac surgery, with incidence rates ranging from 4 to 55% [1, 2]. The etiology involving aspects like neuroinflammation [3] and brain damage [4] remains incompletely understood. Emerging research highlights the importance of blood markers in elucidating POD mechanisms. Biomarkers are known to be closely associated with POD, such as preoperative [5, 6] and postoperative interleukin-6 (IL-6) levels [7], both of which implicate inflammation as one of the mechanisms contributing to POD development [8]. Given the link between IL-6 and POD, we chose to investigate IL-6 as an inflammatory marker. Phosphorylated tau protein 181 (p-tau181) in plasma correlates closely with CSF p-tau181 and is a marker of structural brain damage [9, 10]. In addition, p-tau181 remains stable under the pre-analytical conditions of clinical practice [11] and is therefore suitable for blood sampling in the preoperative clinical setting. Elevated plasma tau (the term “tau” refers here to the middle region of tau as well as to truncated and complete forms of tau) was shown to correlate with delirium severity in POD patients [12] and accompanied by a simultaneous rise in IL-8 and IL-10 [12] linking inflammation to neurodegeneration. While t-tau is a general biomarker for neurodegeneration, p-tau181 is specifically associated with the pathological hallmarks of Alzheimer disease (AD) [13] and POD severity [14]. A recent study showed that preoperative p-tau181 and p-tau217 plasma levels in 491 patients undergoing knee and hip replacement or laminectomy are predictive of POD [15], and of its severity. On the other hand, a recent study demonstrated that the distribution of measured p-tau181 levels in plasma does not differ significantly between patients with POD and those without it [16]. More research is required to clarify this controversy. Severe inflammation is likely to trigger neuronal cell damage and consequently raise the blood concentrations of IL-6 and p-tau181 in POD patients. However, conflicting evidence suggests that higher IL-6 levels correlate with reduced tau pathology in the cerebrospinal fluid [17], indicating a possible neuroprotective role of IL-6. Considering these complexities, our study examines the interplay between heart and brain disease, cognitive functioning, factors related to inflammation (IL-6 levels) such as immunotherapy, and factors related to neurodegeneration (p-tau181 levels) such as cognitive performance in relation to clinical outcomes and pathophysiology of POD following cardiac surgery. We hypothesize that the presence of both biomarkers – p-tau181 and IL-6 – along with preoperative factors related to heart and brain diseases and factors related to the immune system can predict POD in patients undergoing cardiac surgery. This approach postulates that inflammation and neurodegeneration may coexist in POD.

## Methods

### Patient cohort recruitment and preoperative assessments

The FINd DElirium RIsk factors (FINDERI, German Clinical Trials Registration number: DRKS00025095) study is an observational, prospective, and multidisciplinary investigation focused on POD in patients undergoing cardiac surgery [18–20]. Between 2021 and 2022, a total of 571 patients (≥50 years) who consented to take part and underwent elective surgery in the Department of Cardiothoracic and Vascular Surgery at the University Medical Center Göttingen in Germany, were recruited. In addition to age and the requirement for elective surgery, fluency in the German language was defined as an inclusion criterion. Participants with a diagnosis of dementia -documented in the electronic medical records and impairing their ability to follow assessment instructions - were excluded. The Montreal Cognitive Assessment (MoCA) score did not serve as an exclusion criterion, since individuals may score low on the MoCA without meeting the diagnostic criteria for dementia according to the International Classification of Diseases, Tenth Revision (ICD-10). The current analysis centers on investigating the interplay between blood biomarkers such as IL-6 and p-tau181 and POD incidence. In addition, we take into account preexisting heart and brain comorbidities as well immune system factors to enhance the accuracy of POD prediction. We collected data related to heart disease, which includes a history of myocardial infarction, aortic, tricuspid and mitral valve disease and its severity, coronary artery disease, heart failure, the presence of pacemakers or defibrillators, aortic aneurysm, atrial fibrillation, and endocarditis. Factors for brain diseases and mental disorders collected from clinical records and not from those applied for modeling included carotid stenosis, history of cerebral ischemia, dementia, anxiety disorders, addiction, depression, and Parkinson’s disease. Additionally, we examined other somatic factors such as peripheral artery disease, chronic obstructive pulmonary disease, renal insufficiency, recent acute dialysis, cancer within the last 5 years, thyroid disorders, and type 2 diabetes. All these factors were gathered from clinical data as part of the FINDERI study. Our study received ethical approval from the local ethics committee (approval: February 16, 2021, vote: November, 20, 2020). A study protocol was reported previously [18]. The study was in agreement with the Strengthening the Reporting of Observational Studies in Epidemiology (STROBE) Statement [21].

### Fluid biomarkers

#### Processing and storing biomaterial probes

Blood samples were collected 1-2 days before and seven days after cardiac surgery and stored at the biomaterial bank of the Department of Psychiatry and Psychotherapy, University Medical Center Göttingen in Germany. The serum samples underwent a 45-minute incubation period before centrifugation at 2000 x g for 10 minutes at 20°C. Ethylenediaminetetraacetic acid (EDTA) plasma centrifugation was carried out at 2000 x g for 10 minutes. Subsequently, the plasma samples were stored at -80°C in our biomaterial bank.

#### IL-6 measurement

The next generation automated sandwich immunoassay was used to detect IL-6 in human sera samples using Simple-Plex assay on Ella^TM^ (Bio-techne, Minneapolis, USA). The assay was performed according to the manufacturer’s instructions on a single-analyte Ella cartridge. In brief, sera samples were used after 2-fold dilution with sample diluent, and 50 μl of this solution was added to each sample inlet of the Ella cartridge. Subsequently, 1 ml of wash buffer was added to the corresponding wells of the Ella cartridge. The sample results were produced using Simple Plex Runner v.3.7.2.0. The entire procedure took about 90 minutes. The lower limit of quantification (LLOQ) was defined according to the manufacturer. No sample was outside the range of the LLOQ and upper LOQ (ULOQ).

#### Lumipulse based quantification of p-tau181

To determine p-tau181 concentrations, we used commercially available p-tau181 immunoreaction cartridges for plasma on the fully automated Lumipulse G600II system. Quality control measurements were taken. To measure directly EDTA plasma, 400 µL of centrifuged plasma with 2 mL microtubes (Sarstedt, Germany) were added to the device. Simple p-tau181 measurements were taken on the automated Lumipulse device with the same samples. The assays were done as single measurements according to the kit’s instructions. The lower limit of quantification (LLOQ) was defined according to the manufacturer. All samples measured were above the LLOQ.

### Postoperative delirium screening and cognitive assessment

POD screening was conducted twice daily, consisting of morning and evening sessions. These POD assessments involved administration of the Confusion Assessment Method (CAM) for intensive care unit (ICU; CAM-ICU) [22, 23] and I-CAM [24, 25] in the intermediate care unit. The Richmond Agitation Sedation Scale (RASS) [26] was initially performed in the ICU to assess the levels of sedation and agitation. The screening of POD was done by study personnel on the usage of the CAM-ICU and I-CAM, as well as on the Diagnostic and Statistical Manual of Mental Disorders, Fifth Edition (DMS-5) and the ICD-10 diagnostic criteria of delirium. Furthermore, the study staff took part in supervision sessions (weekly or once every two weeks) with a senior consultant (MS) after completing the training [27, 28]. The CAM [22, 24, 25, 29] is a standardized, evidence-based instrument for POD screening. We performed a slightly altered form of the CAM algorithm. In particular, we utilized a short form of CAM [29, 30] by supplementing psychomotor alterations to the CAM algorithm (I-CAM) [24] with the following characteristics as worksheets for the evaluators: altered level of consciousness, inattention, acute onset, disorganized thinking [29], as well as psychomotor alterations. POD existed if the following features were documented [29]: acute change, altered level of consciousness, fluctuation, disorganized thinking or inattention. The psychomotor alterations served to underline the aforementioned POD existence, and to define the POD subtype (e.g., hypoactive or hyperactive POD) [25]. The CAM-ICU exhibits a high sensitivity (0.95-1.00) and a specificity (0.89-0.93) with an inter-rater reliability of 0.88 to 1.0 [22, 24, 25, 29]. The I-CAM has a sensitivity of 0.77 in a geriatric cohort with a high occurrence of dementia and a specificity between 0.96 and 1.00, together with an inter-rater reliability of 0.95 [22, 24, 25, 29]. Furthermore, we have used the MoCA to measure cognition prior to cardiac surgery [31].

### Statistical approach

Statistical analyses were performed using the programming environment R, version 4.3.1 [32]. The planned sample size (*n* = 500) of the FINDERI study was estimated based on the area under the curve (AUC) of a delirium screening tool, considering a dropout rate of 20%. Categorical variables are presented as absolute or relative frequencies (%), while continuous variables are reported using the mean and standard deviation (SD). To compare patients with and without POD, Pearson’s Chi-squared test was utilized for categorical variables, and the Welch Two Sample t-test was used for continuous variables. A two-sided p-value of less than 0.05 was regarded statistically significant. Additionally, univariate logistic regression analysis was conducted to examine relevant somatic factors, with a focus on heart and brain diseases. Biomarkers IL-6 and p-tau181 were log-transformed (10 based log) prior to regression analysis. Odds ratios (ORs) are presented with 95% confidence intervals (CI). For plasma biomarkers p-tau181 and IL-6, individual evaluations as predictors of POD were conducted using Receiver Operating Characteristic (ROC) curves. The AUC is reported with log-transformed 95%-CI [33]. Optimal cut-off values for these biomarkers were determined by simultaneously maximizing specificity and sensitivity, and are reported alongside specificity, sensitivity, negative and positive predictive values [34, 35]. A linear mixed-effects model was applied to log-transformed IL-6 levels, incorporating POD variables, timing of sample collection, and their interaction, while including a random effect for individual patient variation. Furthermore, a multiple logistic regression was conducted with POD as the target variable. This regression included log-transformed preoperative plasma biomarkers and their interactions, along with age, female sex, MoCa results, the presence of tumors, and immunotherapy-related factors identified in the cohort (corticosteroids, colchicine, and cytostatic drugs) as explanatory POD variables. In the logistic regression models, only complete cases are considered. Supplementary Table 1 shows the number of observations for the analyses carried out. Supervised machine learning (ML) methods, specifically regularized logistic regression employing a least absolute shrinkage and selection operator (LASSO) and a classification decision tree, were also utilized to identify key parameters for POD [36, 37]. Input variables included age at baseline, preoperative IL-6 and p-tau181 levels, and somatic conditions. The classification decision tree had a max. depth of four as well as a min. split requirement of 20 observations, determined by the Gini index. LASSO was implemented with prior multiple imputation to address missing and unknown variables. Multiple imputation was conducted five times, with 50 iterations each, using predictive mean matching [38]. The machine learning approach utilized a tenfold cross-validation. A dataset containing 70% of the data was randomly selected as the training set, and the residual 30% of data were utilized for validation [39].

## Results

### Core characteristics of the FINDERI cohort

#### Demographics

A total of 504 patients were included in our cohort analysis (Table 1); 491 of them could be thoroughly evaluated for the presence of POD (Supplementary Table 2). Thirteen patients were excluded from the detailed statistical analysis as a thorough POD assessment was not possible (for dropout analysis see supplementary Table 3). Among them, 106 patients (21.6%) were diagnosed with POD following cardiac surgery, based on the CAM-ICU and I-CAM. Detailed demographic data is presented in Table 1. Patients diagnosed with POD were significantly older than those without POD (mean age with POD: 71.0 ± 7.7 years vs. mean age without POD: 67.6 ± 8.3 years, *p* < 0.001, Table 1). Notable statistical differences were also found between the two groups in terms of MoCA findings, heart failure, implanted pacemaker or defibrillator, heart valve disease, the presence and severity of mitral valve disease, and type 2 diabetes, as detailed in Table 1. POD lasted an average of 3.3 ± 1.3 days, diagnosed 1.4 ± 0.7 days after cardiac surgery and ended 3.8 ± 1.3 days after intervention. Most patients exhibited hypoactive delirium symptoms (45%). A form of mixed POD occurred slightly less frequently (37%); the fewest patients presented with hyperactive POD (18%). POD was diagnosed in 73% of patients via a positive CAM-ICU score and 47% applying the I-CAM), where some patients were positive in both CAM-ICU and I-CAM.

**Table 1:** Characteristics of the total FINDERI cohort and stratified by postoperative delirium: demographics, heart, brain and internal medicine disease factors and plasma biomarker.

Characteristic	*N*	*N* = 504^1^	*N*	no POD, *N* = 385^1^	*N*	POD, *N* = 106^1^	*p*-value^2^
**Sex**	504		385		106		0.078
male		396 (78.57%)		309 (80.26%)		76 (71.70%)	
female		108 (21.43%)		76 (19.74%)		30 (28.30%)	
**Age**	504	68.3 ± 8.2	385	67.6 ± 8.3	106	71.0 ± 7.7	**<0.001**
**BMI**	496	28.3 ± 4.7	382	28.3 ± 4.6	102	28.2 ± 4.7	0.87
**MoCa**	499	23.8 ± 3.6	382	24.3 ± 3.4	104	22.3 ± 4.1	**<0.001**
**Coronary heart disease**	500	368 (73.60%)	382	279 (72%)	105	82 (77%)	0.57
**History of myocardial infarction**	499	126 (25.25%)	381	95 (24.93%)	105	29 (27.62%)	0.67
**Heart failure**	473	365 (77.17%)	363	268 (73.83%)	97	84 (86.60%)	**0.012**
**Implanted pacemaker or defibrillator**	503		384		106		**0.035**
No		482 (95.83%)		373 (97.14%)		98 (92.45%)	
pacemaker		12 (2.39%)		7 (1.82%)		3 (2.83%)	
defibrillator		9 (1.79%)		4 (1.04%)		5 (4.72%)	
**Heart valve disease**	502	318 (63.35%)	383	232 (60.57%)	106	77 (72.64%)	**0.030**
**Aortic valve disease**	501		382		106		0.61
No		294 (58.68%)		225 (58.90%)		61 (57.55%)	
insufficiency		69 (13.77%)		48 (12.57%)		18 (16.98%)	
stenosis		93 (18.56%)		75 (19.63%)		17 (16.04%)	
combined		45 (8.98%)		34 (8.90%)		10 (9.43%)	
**Severity of aortic valve disease**	488		372		104		0.88
none		294 (60.25%)		225 (60.48%)		61 (58.65%)	
mild		46 (9.43%)		33 (8.87%)		12 (11.54%)	
moderate		34 (6.97%)		27 (7.26%)		7 (6.73%)	
severe		114 (23.36%)		87 (23.39%)		24 (23.08%)	
**Mitral valve disease**	502		383		106		**0.012**
No		284 (56.57%)		231 (60.31%)		48 (45.28%)	
insufficiency		207 (41.24%)		146 (38.12%)		53 (50.00%)	
stenosis		5 (1.00%)		2 (0.52%)		3 (2.83%)	
combined		6 (1.20%)		4 (1.04%)		2 (1.89%)	
**Severity of mitral valve disease**	493		381		101		**<0.001**
none		284 (57.61%)		231 (60.63%)		48 (47.52%)	
mild		105 (21.30%)		86 (22.57%)		19 (18.81%)	
moderate		40 (8.11%)		19 (4.99%)		15 (14.85%)	
severe		64 (12.98%)		45 (11.81%)		19 (18.81%)	
**Tricuspid valve disease**	500		381		106		0.10
No		403 (80.60%)		314 (82.41%)		78 (73.58%)	
insufficiency		96 (19.20%)		66 (17.32%)		28 (26.42%)	
stenosis		1 (0.20%)		1 (0.26%)		0 (0.00%)	
combined		0 (0.00%)		0 (0.00%)		0 (0.00%)	
**Severity of tricuspid valve disease**	496		379		104		0.067
none		403 (81.25%)		314 (82.85%)		78 (75.00%)	
mild		66 (13.31%)		48 (12.66%)		18 (17.31%)	
moderate		16 (3.23%)		9 (2.37%)		7 (6.73%)	
severe		11 (2.22%)		8 (2.11%)		1 (0.96%)	
**Aortic aneurysm**	501	40 (7.98%)	383	29 (7.57%)	105	8 (7.62%)	>0.99
**Aortic dissection**	501	4 (0.80%)	383	1 (0.26%)	105	0 (0.00%)	>0.99
**Carotis artery stenosis**	491	69 (14.05%)	377	50 (13.26%)	102	17 (16.67%)	0.47
**Atrial fibrillation**	498	105 (21.08%)	381	73 (19.16%)	104	29 (27.88%)	0.072
**Endocarditis**	497	7 (1.41%)	380	4 (1.05%)	104	3 (2.88%)	0.36
**Peripheral arterial occlusion disease**	500	59 (11.80%)	381	41 (10.76%)	106	17 (16.04%)	0.19
**History of stroke**	499	54 (10.82%)	382	38 (9.95%)	104	15 (14.42%)	0.26
**Renal insufficiency**	502	64 (12.75%)	384	46 (11.98%)	105	17 (16.19%)	0.33
**Acute dialysis requirement**	503	3 (0.60%)	384	3 (0.78%)	106	0 (0.00%)	0.83
**Tumor (during the past 5 years)**	501	45 (8.98%)	383	32 (8.36%)	105	10 (9.52%)	0.86
**Thyroid diseases**	501	87 (17.37%)	382	64 (16.75%)	106	21 (19.81%)	0.56
**Chronic obstructive pulmonary disease**	500	40 (8.00%)	381	31 (8.14%)	106	8 (7.55%)	>0.99
**Diabetes**	503	150 (29.82%)	384	102 (26.56%)	106	46 (43.40%)	**0.001**
**Depressive disorder**	500	61 (12.20%)	382	41 (10.73%)	105	18 (17.14%)	0.11
**Anxiety disorder**	501	23 (4.59%)	383	19 (4.96%)	105	3 (2.86%)	0.51
**Addiction (drugs/alcohol)**	498	16 (3.21%)	381	13 (3.41%)	104	3 (2.88%)	>0.99
**Dementia**	503	1 (0.20%)	384	0 (0.00%)	106	1 (0.94%)	0.49
**Parkinsons disease**	503	2 (0.40%)	384	0 (0.00%)	106	2 (1.89%)	0.066
**Log-transformed preoperative p-tau181**	496	0.4 ± 0.5	379	0.4 ± 0.5	104	0.6 ± 0.5	**<0.001**
**Log-transformed preoperative IL-6**	485	1.6 ± 0.8	375	1.5 ± 0.8	101	1.8 ± 0.8	**0.007**
**Log-transformed postoperative IL-6**	431	3.4 ± 0.7	334	3.3 ± 0.7	91	3.4 ± 0.7	0.71
**Difference of log-transformed IL-6 (post-pre)**	428	1.8 ± 1.0	332	1.8 ± 1.0	90	1.7 ± 0.9	0.26

^1^OR = Odds Ratio.

^2^Pearson’s Chi-squared test or Welch Two Sample t-test.

*n*, number of patients, *BMI* body mass index, *CI* confidence interval, *n* number of patients, *IL*-6 interleukin 6, *MoCa* montreal cognitive assessment, *POD* postoperative delirium, *p-tau* 181 phosphorylated tau protein 181.

#### Preoperative heart and brain disease related factors

We conducted univariate logistic regression analysis to identify significant heart and brain disease related factors associated with the development of POD in those patients assessable for POD (*n* = 491, see supplementary Table 2). The analysis revealed several relevant factors: age (OR 1.05, 95%-CI: 1.02, 1.08, *p* < 0.001), MoCA assessment results (OR 0.86, 95%-CI: 0.81, 0.92, *p* < 0.001), the presence of heart failure (OR 2.29, 95%-CI: 1.26, 4.47, *p* = 0.01), heart valve disease (OR 1.73, 95%-CI: 1.09, 2.81, *p* = 0.024), mitral valve insufficiency (OR 1.75, 95%-CI: 1.12, 2.72, *p* = 0.013), mitral valve stenosis (OR 7.22, 95%-CI: 1.17, 55.9, *p* = 0.033), moderately severe mitral valve disease (OR 3.8, 95%-CI: 1.78, 8.00, *p* < 0.001), extremely severe mitral valve disease (OR 2.03, 95%-CI: 1.08, 3.74, *p* = 0.025), tricuspid valve insufficiency (OR 1.7, 95%-CI: 1.02, 2.82, *p* = 0.038), moderate tricuspid valve disease (OR 3.13, 95%-CI: 1.09, 8.66, *p* = 0.028), and type 2 diabetes (OR 2.12, 95%-CI: 1.35, 3.31, *p* < 0.001). Additionally, the presence of a defibrillator was identified as a significant factor (OR 4.76, 95%-CI: 1.24, 19.5, *p* = 0.022). These findings are presented in supplementary Table 2.

#### Plasma biomarker

Plasma biomarkers p-tau181 and IL-6 were evaluated via univariate regression analysis to determine their significance in predicting POD in our cohort of 491 patients with assessed POD status (Supplementary Table 2). Our analysis indicated that preoperative IL-6 levels represented a significant risk factor (OR 1.38, 95%-CI 1.07, 1.77; *p* = 0.012, *n* = 476), while postoperative IL-6 levels (OR 1.07, 95%-CI 0.77, 1.47; *p* = 0.69, *n* = 425), and the difference between postoperative and preoperative IL-6 levels (OR 0.88, 95%-CI 0.68, 1.12, *p* = 0.29, *n* = 422) did not constitute significant risk factors in a univariate context (Supplementary Table 2). Similarly, preoperative levels of p-tau181 (OR 2.26, 95%-CI 1.46, 3.57; *p* < 0.001, *n* = 483) were found to be a significant predictor of POD in the FINDERI cohort.

### Plasma biomarker to predict postoperative delirium

**Fig. 1:**
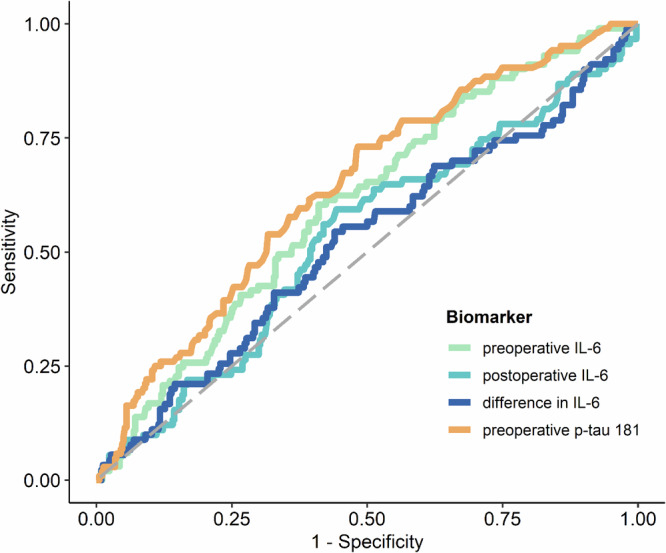
ROC curves of IL-6 and p-tau181 plasma biomarkers for predicting POD. ROC curves of preoperative IL-6 (orange), preoperative p-tau 181 (green), postoperative IL-6 (light blue) and the difference between post- and preoperative IL-6 (dark blue) blood levels for predicting POD following the CAM and CAM-ICU POD definition. Abbreviations: CAM = confusion assessment method, CAM-ICU = Confusion Assessment Method for the Intensive Care Unit, POD = postoperative delirium, IL-6 = interleukin 6, p-tau181 = phosphorylated tau protein 181.

The levels of preoperative IL-6 and preoperative p-tau181 were identified as significant predictors of POD. This is illustrated in Fig. 1; Supplementary Table 4. Supplementary Figure 1 also show the log-transformed biomarker levels of IL-6 and p-tau181. ROC analysis yielded an AUC of 0.605 (95%-CI: 0.544, 0.663, *p* = 0.0018) for preoperative IL-6 levels and an AUC of 0.641 (95%-CI: 0.581, 0.698, *p* < 0.0001) for preoperative p-tau181. The optimal cut-off value for preoperative IL-6 was 4.71, demonstrating a sensitivity of 60.4% and a specificity of 58.9%, as detailed in supplementary Table 5A (for postoperative IL-6 cut off value, see Table 4B). The optimal cut-off value determined for preoperative p-tau 181 was 1.57, with a sensitivity of 61.5% and a specificity 60.7%, as reported in supplementary Table 5C. However, it was observed that POD could not be predicted based on postoperative IL-6 levels or the difference between postoperative and preoperative IL-6 levels, as indicated in supplementary Table 4, supplementary Figures 1B, C and Fig. 1. Furthermore, a linear mixed-effects model for IL-6 demonstrated that while time (*p* < 0.0001) and POD (*p* = 0.03) were significant factors, the interaction between time and POD was not significant (*p* = 0.09), as depicted in supplementary Figure 2. The mean group values in the difference of the log-transformed IL-6 levels for no POD were 1.809 (95%-CI: 1.71, 1.91, *p* < 0.0001) and 1.624 for POD (95%-CI: 1.43, 1.82, *p* < 0.0001).

### Predicting postoperative delirium by combining plasma biomarkers and heart and brain as well as immunotherapy related factors

**Fig. 2:**
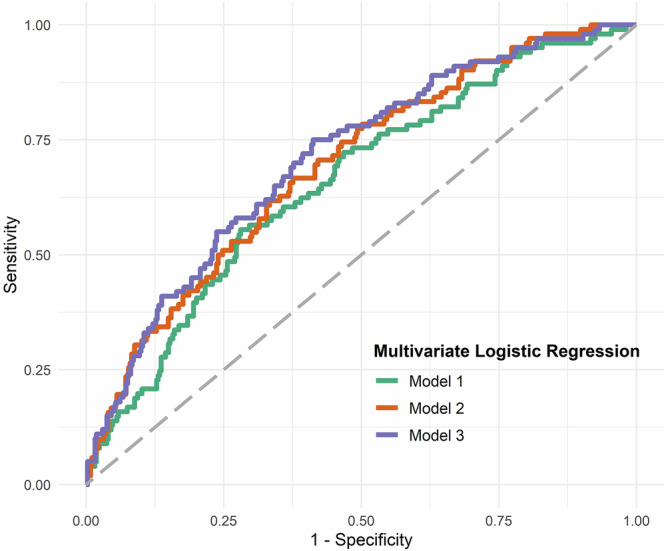
Multiple logistic regression models for predicting POD. Model 1: Plasma log transformed preoperative IL-6 in conjunction with age predict POD with an AUC of 0.658 (95%-CI, 0.596, 0.714, *p* < 0.0001). Model 2: log transformed preoperative IL-6 in conjunction with female sex and cognitive performance (MoCa assessment findings) predict POD with an AUC of 0.694 (95%-CI, 0.637, 0.747, *p* < 0.0001). Model 3: preoperative log-transformed levels of p-tau181 and IL-6, sex and cognitive performance determine POD prediction with an AUC of 0.710 (95%-CI, 0.651, 0.763, *p* < 0.0001). Abbreviation: AUC = area under the curve, CI = confidence interval, MoCa= Montreal Cognitive Assessment, ROC = receiver operating characteristics.

To identify possible associations among biomarkers and various heart and brain as well as immune system related factors, we conducted a multiple logistic regression analysis in three different models (Table 2A). In the first model, IL-6, age, sex, tumor, corticosteroids, colchicine and cytostatic drugs were included as explanatory variables in the analysis; we found that age and log-transformed preoperative IL-6 facilitated the prediction of POD (Table 2B and Fig. 2, Model 1). We investigated the immunotherapy-associated variables in a model with IL-6, since the activity of the inflammatory marker IL-6 can be influenced by immunotherapy. Our first model was able to predict POD with reasonable accuracy, achieving an AUC of 0.658 (95%-CI: 0.596, 0.714, *p* < 0.0001), as shown in Table 2B and Fig. 2, Model 1. Log p-tau181, age, gender, cognitive performance measured by MoCA and education were included as explanatory variables in our second model. It also included cognitive performance together with preoperative p-tau181 levels, as neurodegenerative processes are often associated with reduced cognition. This model revealed that log-transformed preoperative p-tau181, female sex, and cognitive performance were relevant factors. POD could be predicted with this model with moderate accuracy, namely an AUC of 0.694 (95%-CI: 0.637, 0.747, *p* < 0.0001, Table 2C, Fig. 2, Model 2). When we combined all explanatory variables (log-transformed IL-6, log-transformed p-tau181, age, sex, tumor, corticosteroids, colchicine, cytostatic drugs, MoCA findings, education, preoperative log IL-6 and preoperative p-tau 181 and female sex) into one model (Model 3), female sex, education > 10 years and cognitive performance (MoCA) proved to be relevant factors. However, the interaction between the blood biomarkers p-tau181 and IL-6 in blood plasma was not a relevant POD predictor (OR 0.87, 95%-CI: 0.48, 1.56, *p* = 0.63, Table 2, Model 3). With this combined model 3, POD was predictable with moderate accuracy with an AUC of 0.709 (95% CI: 0.651, 0.763, *p* < 0.0001; Table 2D, Fig. 2, Model 3).

**Table 2:** Multiple logistic regression models.

A: Number of observations in the selected multivariate logistic regression models	
Model	Number of observations
Model 1	475
Model 2	477
Model 3	471

B: Model 1			
Characteristic^1^	OR^2^	95% CI^2^	p-value
Log-transformed preoperative IL-6	1.35	1.03, 1.76	0.026
**Age**	1.05	1.02, 1.08	**0.001**
**Sex: female**	1.62	0.95, 2.73	0.071
**Tumor**	1.10	0.48, 2.33	0.82
**Corticosteroids**	1.70	0.63, 4.29	0.27
**Colchicine**	0.00		0.99
**Cytostatics**	0.00		0.99

C: Model 2			
Characteristic^1^	OR^2^	95% CI^2^	p-value
**Log-transformed preoperative p-tau181**	1.63	1.00, 2.65	**0.045**
**Age**	1.02	0.99, 1.05	0.18
**Sex: female**	1.86	1.08, 3.17	**0.024**
**MoCa**	0.87	0.81, 0.94	**<0.001**
**Education**^3^			
Finished secondary general school	0.92	0.25, 3.81	0.90
Finished secondary school	1.06	0.28, 4.55	0.94
Finished polytechnic secondary school	0.93	0.14, 5.76	0.94
Finished vocational school, technical college	1.64	0.30, 9.41	0.57
Finished academic secondary school	1.73	0.41, 8.17	0.47

D: Model 3			
Characteristic^1^	OR^2^	95% CI^2^	p-value
**Log-transformed preoperative IL-6**	1.32	0.85, 2.00	0.20
**Log-transformed preoperative p-tau181**	1.93	0.59, 6.27	0.27
**Age**	1.02	0.99, 1.05	0.18
**Sex: female**	1.93	1.10, 3.34	**0.020**
**Tumor**	1.03	0.43, 2.26	0.95
**Corticosteroids**	1.96	0.69, 5.21	0.19
**Colchicine**	0.00		0.99
**Cytostatics**	0.00		0.99
**MoCa**	0.87	0.81, 0.94	**<0.001**
**Education**^3^			
Finished secondary general school	1.05	0.27, 4.60	0.94
Finished secondary school	1.17	0.29, 5.30	0.83
Finished polytechnic secondary school	1.25	0.18, 8.23	0.82
Finished vocational school, technical college	2.37	0.40, 14.7	0.34
Finished academic secondary school	2.19	0.49, 11.1	0.32
Log-transformed preoperative IL-6* Log-transformed preoperative p-tau181	0.87	0.48, 1.56	0.63

^1^AUC 0.6575, (95%-CI 0.5957, 0.7138; *p* < 0.0001),

^2^*OR* odds ratio, *CI* confidence interval.

IL-6 interleukin 6

^1^AUC 0.6939, (95%-CI 0.6365, 0.7463; *p* < 0.0001), ^2^OR = Odds Ratio, CI = Confidence Interval,

^3^The German secondary education system with secondary educational institutions in Germany is depicted in supplementary table 7.

*MoCa* montreal cognitive assessment, *p-tau*181 = phosphorylated tau protein 181

^1^AUC 0.7085, (95%-CI 0.6492, 0.7619; *p* < 0.0001), ^2^*OR* odds ratio, *CI* confidence interval,

^3^The German secondary education system with secondary educational institutions in Germany is depicted in supplementary table 7.

IL-6 = interleukin 6, MoCa = Montreal Cognitive Assessment, p-tau181 = phosphorylated tau protein 181

### Predicting postoperative delirium taking a machine learning approach

#### Decision tree

**Fig. 3:**
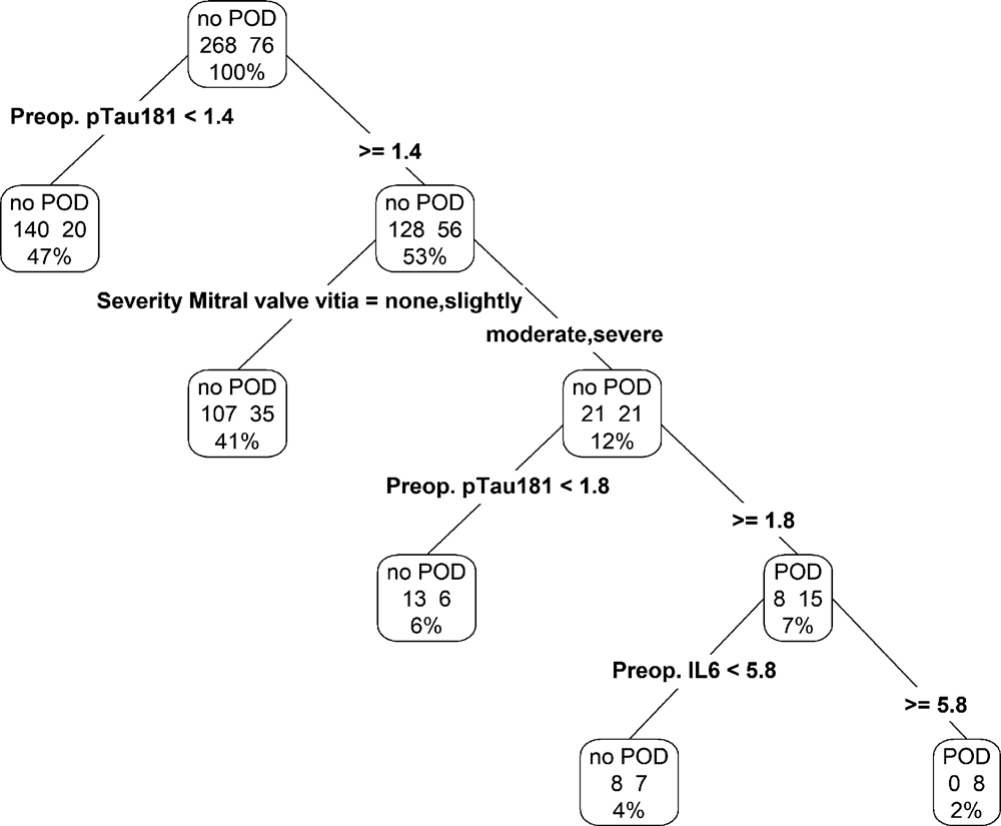
Decision tree for POD prediction. Decision tree with the four most predictive variables for POD, ie, preoperative p-tau181 value, preoperative IL-6 and severity of mitral valve disease. Abbreviation: POD = postoperative delirium, p-tau 181 = phosphorylated tau protein 181, IL-6 = interleukin 6.

To identify the most important guidelines for predicting POD, we created a decision showing four important rules. The rules were applied in the following sequence: (1) preoperative p-tau181 level greater than 1.4, (2) the presence of moderate or severe mitral valve disease, (3) a preoperative p-tau181 exceeding 1.8, and (4) preoperative IL-6 level above 5.8, as depicted in Fig. 3. The performance of this decision tree model was quantitatively evaluated, showing an AUC of 0.672 (95%-CI: 0.604, 0.735, *p* < 0.0001) on the training set and 0.642 (95%-CI: 0.537, 0.738, *p* = 0.0108) on the validation set. These results, along with detailed visual representations, are provided in supplementary Table 6, Fig. 3.

#### LASSO

**Fig. 4:**
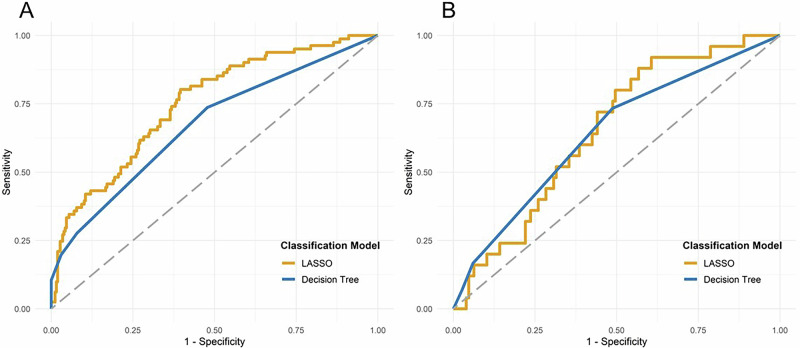
ROC curves of the classification models regularized LASSO and decision trees. ROC analysis revealed that the classification model decision tree yielded significantly predictive accuracy of POD for the training (**A**) and validation (**B**) datasets [A, B: *p* < 0.05; AUC for the training dataset of 0.672 (95%-CI: 0.604, 0.735, *p* < 0.0001); AUC for the validation dataset of 0.642 (95%-CI: 0.537, 0.738, *p* = 0. 0108)]. LASSO also predicted POD accurately with an AUC of 0.751 (95%-CI: 0.686, 0.805, *p* < 0.0001) for the training dataset (**A**) and 0.652 (95%-CI: 0.538, 0.747, *p* = 0.0086) for the validation dataset (**B**). Abbreviations: CI = confidence interval, ROC = receiver operating characteristics, POD = postoperative delirium.

During the application of the LASSO machine learning procedure, non-zero regression coefficient estimates were observed for two variables: age and preoperative p-tau181 levels. The performance of the LASSO model in predicting POD was assessed using ROC analysis, which demonstrated an AUC of 0.751 (95%-CI: 0.686, 0.805, *p* < 0.0001) for the training and an AUC of 0.652 (95%-CI: 0.539, 0.747, *p* = 0.0086) for the validation set, reflecting a moderate level of prediction accuracy. These findings, along with the corresponding graphical representation, are detailed in supplementary Table 6 and Fig. 4A, B.

## Discussion

Our research in a substantially large cohort reveals that measuring preoperative p-tau181 and IL-6 levels in blood plasma in conjunction with mitral valve disease, cognitive performance, female sex, and age are important to enable the prediction of POD in patients undergoing cardiac surgery. This insight carries significant potential clinical relevance even though some factors such as mitral valve disease are known to be independent predictors of poor outcome [40]. A data-driven ML approach utilizing decision trees was instrumental in identifying specific assessment criteria. These criteria, which include preoperative p-tau181 and IL-6 levels, as well as severe mitral valve disease, proved effective in predicting POD. Moreover, incorporating these blood biomarkers and human disease factors related to heart and brain diseases into a LASSO procedure yielded the highest accuracy in our analysis with an AUC of 0.751 in the training set, although the calculation in the validation set could not uphold the same AUC level with an AUC of 0.652, indicating a certain degree of overfitting. To date, no other study has explored the combined analysis of p-tau181 and IL-6 levels along with a broad range of heart and brain related factors as predictors of POD. Consequently, our predictive model entailing specific p-tau181 and IL-6 cut-off levels within the decision tree stands as a unique contribution to this field, offering a novel perspective in the assessment and management of POD risk.

### Biomarker p-tau181 for predicting postoperative delirium

In our study, we explored the potential of p-tau181 levels as a blood biomarker for predicting POD. We observed a significant predictive capability with an AUC of 0.641 in the ROC analysis. However, studies investigating p-tau181’s role in predicting non-cardiac POD showed a considerably higher AUC of 0.885 [15] and 0.869 [41]. This notable difference in AUC may be attributed to technical variations in the assays used: Liang et al. utilized established nanoneedle technology, whereas our study employed an immunoassay approach with p-tau181 measurement using Fujirebio’s Lumipulse device.

While the sample sizes in both studies were relatively comparable, the type of surgery (cardiac vs. hip vs. laparoscopic) differed, potentially contributing to the observed discrepancies in accurately diagnosing POD. Another recent study by Leung et al. [16] identified significant difference in p-tau181 levels between POD and non-POD patients. However, their study did not find a significant difference in distribution of p-tau181 levels between these groups in a Kolomorogov-Smirnov analysis. This contrasts with our findings and those of Liang et al. [15], possibly due to the fact that Leung et al. [16] examined a distinct population not undergoing cardiac surgery. Our investigation into the role of p-tau181 levels as a marker for POD in the context of cardiac surgery is novel, underscoring the biomarker’s potential importance in predicting susceptibility to neurodegeneration associated with POD. In addition, cognitive performance and female sex should be considered as factors for predicting POD when p-tau181 blood plasma levels serve as a blood biomarker-based strategy for predicting POD.

### Blood IL-6 as a postoperative delirium predictor

Our study’s findings regarding the prediction of POD using preoperative versus postoperative IL-6 levels are in alignment with those from numerous other studies [5–7, 42–46]. Specifically, we validated the use of pre- and postoperative IL-6 levels as predictors of POD, obtaining similar to slightly lower AUC values in the ROC analysis, with preoperative IL-6 at 0.73 and postoperative IL-6 at 0.72 in patients undergoing cardiac surgery [5]. Our analysis revealed that preoperative IL-6 levels play a particularly significant role in predicting POD, with an AUC of 0.605 in the ROC curve. Recent research suggests that the soluble ectodomain of trigger receptor 2 (sTREM2) expressed on myeloid cells, which plays an important role in neuroglial inflammation, is modulated by IL-6 levels, which are already elevated in POD patients on the first postoperative day [47], thus revealing a key indication of a pathophysiological link between possible glial neuroinflammation and inflammation detectable in the blood via a rise in the proinflammatory marker IL-6. Moreover, IL-6 levels have been observed to rise steadily during initial hours following a coronary artery bypass graft surgery. Particularly, an IL-6 level exceeding a value of 583 pg/ ml after the 18th postoperative hour was associated with an increased incidence of POD [43]. Our results, therefore, suggest the need for a more nuanced combination of blood biomarkers to improve the accuracy of POD prediction, as indicated by the outcomes of our ROC analysis. It is also important to consider age as a factor in predicting POD when relying on the blood biomarker IL-6 to predict delirium.

### Combining plasma biomarkers with other factors to predict POD

The observed elevated postoperative IL-6 levels in patients with POD underscores the critical role of ongoing inflammation in the development of POD. This connection was recently confirmed in a study by Vasunilashorn et al. [48], who identified inflammation through different proteomics as a common pathway of progression and risk for POD. Complementing this perspective, a study by Liu et al. [42] reported that patients experiencing delirium showed increases in plasma neurofilament light chains and glial fibrillary acid protein, markers indicative of axonal injury and glial inflammation, a pattern not seen in non-delirium patients. The novelty of our study is not that we investigated the general neuroinflammatory marker IL-6, but that we combined it with a cell destruction marker and the link between increased neuroinflammation associated with POD and subsequent axonal brain damage. To our knowledge, no prior study has integrated such biomarkers. Although our ROC analysis showed only a modest increase in the AUC (0.671) compared to studies focusing solely on IL-6 and p-tau181 biomarkers, this outcome may be partly attributed to the significant influence of age, as revealed by the logistic regression analysis. Future research should therefore include subgroup analyses of potential patients undergoing heart surgery, particularly focusing on the incidence of POD. Such studies could identify patient subpopulations that might benefit most from POD prediction using these combined biomarkers. Taken together, we demonstrate that patients who developed POD presented higher preoperative plasma concentrations of IL-6 or p-tau181 than those who did not develop POD. The preoperative plasma concentration of Il-6 or p-tau181 predicted the presence and severity of POD, with p-tau181 being most closely associated with that outcome. Our multiple logistic regression model further revealed female sex as a relevant POD-predicting factor in this context. Therefore, female gender should be included in predicting delirium in conjunction with the plasma biomarkers IL-6 and p-tau181 in future studies.

### Age, cognitive performance, and blood biomarkers predicting POD

Previous research suggests that both age and preoperative cognitive status are risk factors for the development of POD [49–51], but few studies have examined these relationships while accounting for the effects of inflammation and neuronal cell injury. In our analyses, age was associated significantly with POD in Model 1 (when IL-6 was included in the model), but not in Model 2 (when adjusted for p-tau181) or Model 3 (when adjusted for IL-6, ptau181, and MoCA). These results suggest that age is related to the POD risk even when accounting for preoperative inflammation (as determined by blood IL-6 levels; Model 1). Cognitive performance (assessed by MoCA score), in contrast, emerged as a particularly relevant risk factor when considering the presence of preoperative neuronal injury (p-tau181, Model 2) and both inflammatory and neurodegenerative markers simultaneously (Model 3). While both age and cognitive performance are associated with POD risk, our analyses also indicate that the contribution of these factors is less pronounced than those of inflammation (IL-6, Model 1: OR 1.35) and neurodegeneration (p-tau181, Model 2: OR 1.63). These findings are in line with previous studies [50, 51] confirming that both age and cognitive status are important risk factors for POD, but they also highlight that their effects should be interpreted in the context of biological markers of inflammation and neurodegeneration. Notably, another study confirmed - consistent with Model 2 - that preoperative plasma p-tau181 is an age-independent predictor of POD [41]. The odds ratios suggest that pre-existing neuronal cell damage (p-tau181, OR = 1.63, Model 2) may be a more decisive factor for POD than preoperative inflammation (IL-6, OR = 1.35, Model 1).

### Limitations

Despite the fact that all our study participants were undergoing cardiac surgery, our cohort was quite heterogeneous. This diversity stemmed from a variety of heart surgeries performed and the broad range of ages among patients. This heterogeneity required us to account for a multitude of somatic factors that were evidently influential in the patients’ need for cardiac surgery. Furthermore, the study was conducted at a single center, which may have caused institutional bias. These limitations underscore the need for further research, possibly enhancing the predictive accuracy for POD. Another limitation concerns age-related cognitive decline, dementia, and neurodegeneration as potential confounders of POD. To address this, we incorporated age into our multiple regression analyses, lowered the minimum inclusion age to 50 years, and excluded individuals with a preoperative diagnosis of dementia or neurodegeneration. Overfitting is another problem that must be considered when selecting predictors [52]. The LASSO procedure enforces sparsity of the mode and reduces the number of covariables as a result. By applying a LASSO procedure in combination with cross-validation, overfitting can be reduced. This can ultimately increase the generalizability of our conclusions.

## Conclusions

In this novel analysis, which included preoperative cardiac factors and markers of inflammation and neurodegeneration, we found that a combination of plasma biomarkers (p-tau181 and IL-6) along with specific heart related factors such as mitral valve disease, cognitive performance, age, and female sex can help predict the occurrence of POD in patients undergoing cardiac surgery. Our data suggest that pre-existing neuronal injury may be a slightly stronger predictor of POD than preoperative inflammation. Furthermore, our findings indicate that the interaction between preoperative neuronal injury and inflammation does not appear to contribute meaningfully to POD risk. ML approaches, such as decision trees and LASSO, offer additional tools for predicting and understanding the risk factors associated with POD. Our findings emphasize the importance of taking a comprehensive approach to assess and manage the POD risk in this patient population. The associations and predictors of POD we identified provide a foundation for further research in this field. Confirming these associations and delving into the underlying mechanisms will contribute to a deeper understanding of how POD manifests in the context of cardiac surgery. Such research can pave the way for developing more effective interventions for preventing or managing POD in this specific patient population. This might have potential clinical relevance in identifying patients carrying a higher risk for POD before surgery, aiming to prevent life-threatening complications.

## Supplementary information

Supplementary material

## Data Availability

The FINDERI dataset generated and/or analyzed for the present article is available upon request to the corresponding author. The investigators follow the FAIR Principles for scientific data management and stewardship.
