# Supplementary material for: Prediction of postoperative delirium after cardiac surgery by the interplay between preoperative plasma p-tau181 and IL-6 and heart-brain axis related factors: results from the prospective observational study FINDERI
Source: Mol Psychiatry. 2025 Dec 22;31(5):2509–19. doi: 10.1038/s41380-025-03412-3 (PMC13099388; doi:10.1038/s41380-025-03412-3)

Supplementary Tables

Supplementary Table 1. Number of observations of the analyses

AUC preoperative IL-6	476
AUC postperative IL-6	425
AUC IL-6 difference	422
AUC preoperative p-tau181	483
Linear mixed model for IL-6	901 observations from 479 patients
Logistic regression (combination of the biomarkers)	475

Abbreviations: AUC = area under the curve, IL-6 = interleukin 6, p-tau181 = phosphorylated tau protein 181

Supplementary Table 2. Univariate logistic regression analysis of the FINDERI cohort: demographics, heart, brain and internal medicine disease factors and plasma biomarkers

Characteristic	N	OR[1]	95% CI[1]	p-value
**Sex**	491			
female		1.60	0.97, 2.61	0.059
**Age**	491	1.05	1.02, 1.08	**<0.001**
**BMI**	484	1.00	0.95, 1.04	0.87
**MoCa**	486	0.86	0.81, 0.92	**<0.001**
**Coronary heart disease**	487			
Yes		1.32	0.80, 2.24	0.30
**History of myocardial infarction**	486			
Yes		1.15	0.70, 1.85	0.58
**Heart failure**	460			
Yes		2.29	1.26, 4.47	**0.010**
**Implanted pacemaker or defibrillator**	490			
pacemaker		1.63	0.35, 5.98	0.48
defibrillator		4.76	1.24, 19.5	**0.022**
**Heart valve disease**	489			
Yes		1.73	1.09, 2.81	**0.024**
**Aortic valve disease**	488			
insufficiency		1.38	0.74, 2.52	0.30
stenosis		0.84	0.45, 1.49	0.56
combined		1.08	0.48, 2.25	0.83

Characteristic	N	OR[1]	95% CI[1]	p-value
**Severity aortic valve disease**	476			
mild		1.34	0.63, 2.69	0.42
moderate		0.96	0.37, 2.19	0.92
severe		1.02	0.59, 1.72	0.95
**Mitral valve disease**	489			
insufficiency		1.75	1.12, 2.72	**0.013**
stenosis		7.22	1.17, 55.9	**0.033**
combined		2.41	0.33, 12.7	0.32
**Severity mitral valve disease**	482			
mild		1.06	0.58, 1.89	0.84
moderate		3.80	1.78, 8.00	**<0.001**
severe		2.03	1.08, 3.74	**0.025**
**Tricuspid valve disease**	487			
insufficiency		1.71	1.02, 2.82	**0.038**
stenosis		0.00		0.98
**Severity tricuspid valve disease**	483			
mild		1.51	0.82, 2.70	0.18
moderate		3.13	1.09, 8.66	**0.028**
severe		0.50	0.03, 2.80	0.52
**Aortic aneurysm**	488			
Yes		1.01	0.42, 2.18	0.99
**Aortic dissection**	488			
Yes		0.00		0.98
**Carotis artery stenosis**	479			
Yes		1.31	0.70, 2.34	0.38
**Atrial fibrillation**	485			
Yes		1.63	0.98, 2.67	0.054
**Endocarditis**	484			
Yes		2.79	0.54, 12.9	0.18
**Peripheral arterial occlusion disease**	487			
Yes		1.58	0.84, 2.88	0.14
**History of stroke**	486			
Yes		1.53	0.78, 2.85	0.20
**Renal insufficiency**	489			
Yes		1.42	0.76, 2.55	0.26
**Acute dialysis requirement**	490			
Yes		0.00		0.98
**Tumor (during the past 5 years)**	488			

Characteristic	N	OR[1]	95% CI[1]	p-value
Yes		1.15	0.52, 2.36	0.71
**Thyroid diseases**	488			
Yes		1.23	0.70, 2.10	0.46
**COPD**	487			
Yes		0.92	0.38, 1.98	0.84
**Diabetes**	490			
Yes		2.12	1.35, 3.31	**<0.001**
**Depressive disorder**	487			
Yes		1.72	0.92, 3.10	0.077
**Anxiety disorder**	488			
Yes		0.56	0.13, 1.69	0.36
**Addiction (drugs/alcohol)**	485			
Yes		0.84	0.19, 2.67	0.79
**Dementia**	490			
Yes		2,849,770	0.00, NA	0.98
**Parkinsons disease**	490			
Yes		7,820,972	0.00, NA	0.98
**Log-transformed preoperative p-tau181**	483	2.26	1.46, 3.57	**<0.001**
**Log-transformed preoperative IL6**	476	1.38	1.07, 1.77	**0.012**
**Log-transformed postoperative IL6**	425	1.07	0.77, 1.47	0.69
**Difference of log-transformed IL6 (post-pre)**	422	0.88	0.68, 1.12	0.29

[1]OR = Odds Ratio, CI = Confidence Interval, BMI = Body mass index, IL-6 = Interleukin 6, p-tau 181 = phosphorylated tau protein 181, MoCa = Montreal Cognitive Assessment

Supplementary Table 3. Drop out analysis of the total FINDERI cohort: demographics, heart, brain and internal medicine disease factors and plasma biomarker

Characteristic	N	POD assessable, N = 491[1]	N	POD not assessable, N = 13[1]	N	P-value
**Sex**	504					0.84
male		385 (78.41%)		11 (84.62%)		
female		106 (21.59%)		2 (15.38%)		
**Age**	504	68.3 ± 8.3		67.3 ± 6.5		0.58
**BMI**	496	28.3 ± 4.6	278	28.6 ± 7.9	12	0.89
**MoCa**	499	23.8 ± 3.6	486	23.5 ± 3.9	13	0.74

Characteristic	N	POD assessable, N = 491[1]	N	POD not assessable, N = 13[1]	N	P-value
**Coronary heart disease**	500	361 (74.13%)	487	7 (53.85%)	13	0.19
**History of myocardial infarction**	499	124 (25.51%)	486	2 (15.38%)	13	0.61
**Heart failure**	473	352 (76.52%)	460	13 (100.00%)	13	0.10
**Implanted pacemaker or defibrillator**	503		490		13	**0.007**
No		471 (96.12%)		11 (84.62%)		
pacemaker		10 (2.04%)		2 (15.38%)		
defibrillator		9 (1.84%)		0 (0.00%)		
**Heart valve disease**	502	309 (63.19%)	489	9 (69.23%)	13	0.88
**Aortic valve disease**	501		488		13	0.63
No		286 (58.61%)		8 (61.54%)		
insufficiency		66 (13.52%)		3 (23.08%)		
stenosis		92 (18.85%)		1 (7.69%)		
combined		44 (9.02%)		1 (7.69%)		
**Severity aortic valve disease**	488		476		12	0.81
none		286 (60.08%)		8 (66.67%)		
mild		45 (9.45%)		1 (8.33%)		
moderate		34 (7.14%)		0 (0.00%)		
severe		111 (23.32%)		3 (25.00%)		
**Mitral valve disease**	502		489		13	0.49
No		279 (57.06%)		5 (38.46%)		
insufficiency		199 (40.70%)		8 (61.54%)		
stenosis		5 (1.02%)		0 (0.00%)		
combined		6 (1.23%)		0 (0.00%)		
**Severity mitral valve disease**	493		483		11	**<0.001**
none		279 (57.88%)		5 (45.45%)		
mild		105 (21.78%)		0 (0.00%)		
moderate		34 (7.05%)		6 (54.55%)		
severe		64 (13.28%)		0 (0.00%)		
**Tricuspid valve disease**	500		487		13	0.93
No		392 (80.49%)		11 (84.62%)		
insufficiency		94 (19.30%)		2 (15.38%)		
stenosis		1 (0.21%)		0 (0.00%)		
combined		0 (0.00%)		0 (0.00%)		
**Severity tricuspid valve disease**	496		483		13	**0.005**

Characteristic	N	POD assessable, N = 491[1]	N	POD not assessable, N = 13[1]	N	P-value
none		392 (81.16%)		11 (84.62%)		
mild		66 (13.66%)		0 (0.00%)		
moderate		16 (3.31%)		0 (0.00%)		
severe		9 (1.86%)		2 (15.38%)		
**Aortic aneurysm**	501	37 (7.58%)	489	3 (23.08%)	13	0.13
**Aortic dissection**	501	1 (0.20%)	488	3 (23.08%)	13	**<0.001**
**Carotis artery stenosis**	491	67 (13.99%)	479	2 (16.67%)	12	>0.99
**Atrial fibrillation**	498	102 (21.03%)	485	3 (23.08%)	13	>0.99
**Endocarditis**	497	7 (1.45%)	484	0 (0.00%)	13	>0.99
**Peripheral aterial occlusion disease**	500	58 (11.91%)	487	1 (7.69%)	13	0.98
**History of stroke**	499	53 (10.91%)	486	1 (7.69%)	13	>0.99
**Renal insufficiency**	502	63 (12.88%)	489	1 (7.69%)	13	0.89
**Acute dialysis requirement**	503	3 (0.61%)	488	0 (0.00%)	13	>0.99
**Tumor diseases (during the past 5 years)**	501	42 (8.61%)	488	3 (23.08%)	13	0.19
**Thyroid diseases**	501	85 (17.42%)	488	2 (15.38%)	13	>0.99
**COPD**	500	39 (8.01%)	487	1 (7.69%)	13	>0.99
**Diabetes mellitus**	503	148 (30.20%)	490	2 (15.38%)	13	0.40
**Depressive disorder**	500	59 (12.11%)	487	2 (15.38%)	13	>0.99
**Anxiety disorder**	501	22 (4.51%)	488	1 (7.69%)	13	>0.99
**Addiction (drugs/alcohol)**	498	16 (3.30%)	485	0 (0.00%)	13	>0.99
**Dementia**	503	1 (0.20%)	490	0 (0.00%)	13	>0.99
**Parkinsons disease**	503	2 (0.41%)	490	0 (0.00%)	13	>0.99
**Log-transformed preoperative ptau181**	496	0.4 ± 0.5	483	0.4 ± 0.5	13	0.74
**Log-transformed preoperative IL6**	485	1.6 ± 0.8	476	1.5 ± 0.6	9	0.81
**Log-transformed postoperative IL6**	431	3.3 ± 0.7	425	4.4 ± 1.1	4	0.069
**Difference of log-transformed IL6 (post-pre)**	428	1.8 ± 1.0	422	3.0 ± 1.4	4	0.094

[1]n (%); Mean ± SD. Abbreviations: BMI = body mass index, COPD = chronic obstructive pulmonary disease, IL-6 = interleukin 6, MoCa = Montreal Cognitive Assessment, N = number, POD = postoperative delirium, p-tau181 = phosphorylated tau protein 181, sec = seconds, TMTA = Trial Making Test A, TMTB = Trial Making Test B

Supplementary Table 4: Receiver Operating Characteristic (ROC) analysis of biomarkers for prediction of POD according to CAM-ICU and I-CAM

AUC of IL-6 and p-tau181

	Estimator	Statistic	Lower 95%-CI	Upper 95%-CI	p-value
Preoperative IL-6	0.6054	3.3576	0.5437	0.6636	0.0018
Postoperative IL-6	0.5315	0.9013	0.4638	0.5987	0.3668
Difference IL-6 (post-pre)	0.5206	0.5764	0.4510	0.5881	0.5522
Preoperative p-tau181	0.6411	4.4796	0.5806	0.6975	<0.0001

Abbreviations: AUC = are under the curve, CI = confidence interval (logit-transformed, permutation based), CAM = Confusion Assessment Method, CAM-ICU=Confusion Assessment Method for the intensive care unit, IL-6 = interleukin 6, p-tau181 = phosphorylated tau protein 181

Supplementary Table 5. Prognostic accuracy at optimal cut-off values for the blood biomarkers IL-6 and p-tau181

A. Prognostic accuracy at optimal cut-off value 4.71 for preoperative IL-6

statistic	estimator	lower 95%-CI	upper 95%-CI
Sensitivity	0.6040	0.5017	0.6999
Specificity	0.5893	0.5377	0.6396
PPV	0.2837	0.2245	0.3490
NPV	0.8467	0.7972	0.8882

B. Prognostic accuracy at optimal cut-off value 27.5 for postoperative IL-6

statistic	estimator	lower 95%-CI	upper 95%-CI
Sensitivity	0.5714	0.4634	0.6747
Specificity	0.5629	0.5078	0.6168
PPV	0.2626	0.2028	0.3297
NPV	0.8282	0.7727	0.8749

C. Prognostic accuracy at optimal cut-off value 1.57 for preoperative p-tau181

statistic	estimator	lower 95%-CI	upper 95%-CI
Sensitivity	0.6154	0.5149	0.7091
Specificity	0.6069	0.5557	0.6564
PPV	0.3005	0.2397	0.3669
NPV	0.8519	0.8038	0.8920

Abbreviations: CI = confidence interval (exact binomial confidence limits), PPV = positive predictive value, NPV = negative predictive value, IL-6 = interleukin-6, ptau-181 = phosphorylated tau protein 181.

Supplementary Table 6. Machine learning model performances in POD prediction in training and validation for regularized LASSO multiple regression and decision tree

	AUC	AUC lower 95%-CI	AUC upper 95%-CI	AUC. p-value	Accuracy	Accuracy.lower 95%-CI	Accuracy.upper 95%-CI	Sensitivity	Specificity	PPV	NPV
LASSO: Training	0.7505	0.6861	0.8051	<0.0001	0.7611	0.7120	0.8055	0.0247	0.9922	0.50	0.7642
Decision Tree: Training	0.6719	0.6042	0.7353	<0.0001	0.8023	0.7563	0.8431	0.1053	1.0000	1.00	0.7976
LASSO: Validation	0.6517	0.5385	0.7472	0.0086	0.8158	0.7449	0.8740	0.0000	0.9764	0.00	0.8322
Decision Tree: Validation	0.6415	0.5371	0.7384	0.0108	0.7891	0.7142	0.8520	0.0667	0.9744	0.40	0.8028

Abbreviations: AUC = are under the curve, CI = confidence interval (log-transformed, permutation based), PPV = positive predictive value, NPV = negative predictive value.

Supplementary Table 7: The German secondary education system with secondary education institutions in Germany

<table>
<tr><td>Grade</td><td></td><td></td><td></td><td></td><td>Age</td></tr>
<tr><td>13</td><td rowspan="9">Academic secondary school = Gymnasium</td><td rowspan="3">Vocational school, technical college</td><td rowspan="3">Vocational school, technical college</td><td></td><td>18/19</td></tr>
<tr><td>12</td><td></td><td>17</td></tr>
<tr><td>11</td><td></td><td>16</td></tr>
<tr><td>10</td><td>Tenth year of school</td><td rowspan="6">Secondary school = Realschule</td><td rowspan="6">Polytechnic secondary school = Polytechnische Oberschule, School system in GDR</td><td>15</td></tr>
<tr><td>9</td><td rowspan="5">Secondary general school = Hauptschule</td><td>14</td></tr>
<tr><td>8</td><td>13</td></tr>
<tr><td>7</td><td>12</td></tr>
<tr><td>6</td><td>11</td></tr>
<tr><td>5</td><td>10</td></tr>
</table>

The table is modified from [https://www.studying-in-germany.org/german-education-system/#primary-education. Access date 27.06.2025]. Abbreviation: GDR = German Democratic Republic

Brief description of the German education system at secondary level

The classification of education refers to the German school and education system. In the following, the terms in Table 2 are briefly explained for the classification of the different school pathways. All terms used refer to the secondary school level after primary general school. **Secondary general school** (Hauptschule) corresponds to school education after primary school in Germany from 5 to 9 grade (Klasse) with the secondary general leaving certificate with a usual proportion of 10-14 years (Alter). In addition, there is the **secondary school (Realschule)**, which comprises grades 5 to 10 and is usually attended between the ages of 10 and 15 and concludes with the secondary school leaving certificate. The **polytechnic secondary school** (Polytechnische Oberschule, Schulform der DDR) can also be attended as a special type of secondary school, which also comprises grades 5 to 10 and concludes with the leaving certificate of the polytechnic secondary school. These types of school are also referred to as lower secondary level. A qualification from the lower secondary level qualifies for vocational training at the upper secondary level. The upper secondary level includes the **vocational school, technical college (Berufsschule, Fachoberschule)**, which covers grades 11 to 13 (Klasse) and is usually attended between the ages of 16 and 18 or 19 and concludes with a certificate. Finally, there is the **academic secondary school** (Gymnasium), which covers grades 5 to 13 and is usually attended between the ages of 10 and 18 or 19 (Alter) and concludes with the academic secondary leaving certificate (Abitur). The academic secondary school includes the secondary lower and upper levels.

Supplementary Figures

Supplementary Figure 1. Differences between IL-6 and p-tau181 plasma biomarkers between POD and non-POD patients

Boxplots for POD and non-POD patients of preoperative IL-6 (A), postoperative IL-6 (B), difference between preoperative and postoperative IL-6 (C) and preoperative p-tau 181 (D) blood levels applying the CAM and CAM-ICU POD definitions. Abbreviations: CAM = confusion assessment method, CAM-ICU = Confusion Assessment Method for the Intensive Care Unit, POD = postoperative delirium, IL-6 = interleukin 6, p-tau181 = phosphorylated tau protein 181.

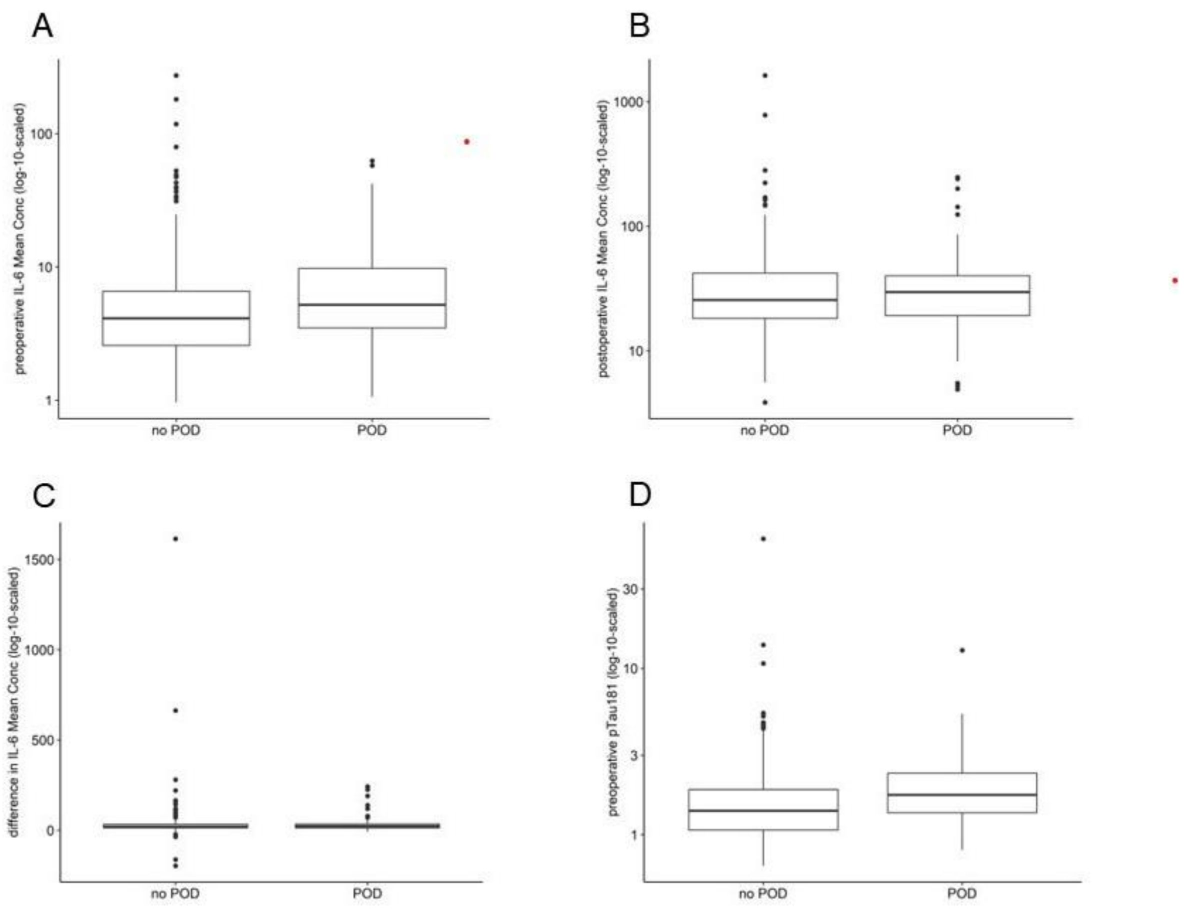

Supplementary Figure 2. Linear mixed effect model for IL-6 between pre- and postoperative blood levels in POD and non-POD patients

Linear mixed effect model analysis for IL-6 showed that time and POD were significant factors, but not the interaction between POD and time (A). Abbreviation: POD = postoperative delirium.

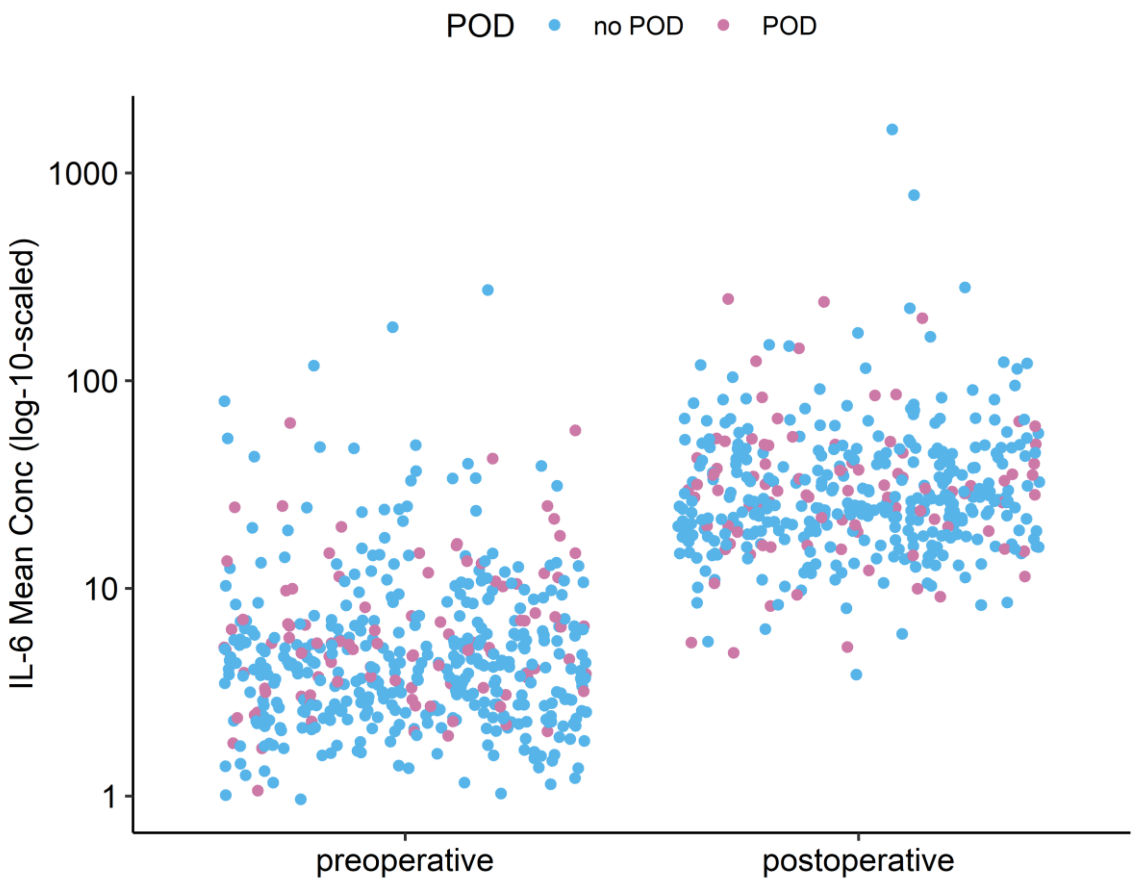

Supplement: Supplementary file 1 — Supplementary material [file 41380_2025_3412_MOESM1_ESM.pdf]
